# Association between the histopathologic measurement of tumor–visceral peritoneal distance and prognosis in T3 colon adenocarcinoma

**DOI:** 10.3389/pore.2026.1612480

**Published:** 2026-07-13

**Authors:** Özgecan Hamdemir, Semin Ayhan, Ömer Atmış, Ömer Acar

**Affiliations:** 1 Department of Pathology, Kafkas University Faculty of Medicine, Kars, Türkiye; 2 Department of Pathology, Manisa Celal Bayar University Faculty of Medicine, Manisa, Türkiye; 3 Clinical Oncology, Kızıltepe State Hospital, Mardin, Türkiye

**Keywords:** colonic neoplasms, lymphatic metastasis, neoplasm invasiveness, perineural invasion, survival analysis

## Abstract

**Objective:**

To investigate the relationship between microscopic tumor–visceral peritoneal distance (T-VPD) and histopathological markers of aggressiveness in pT3 colon adenocarcinoma and to assess its prognostic significance.

**Methods:**

A total of 329 resection specimens were retrospectively analyzed. T-VPD was defined as the shortest distance between the deepest invasive tumor front and the true serosal surface. Cut-off values of ≤0.01 cm, ≤0.05 cm, ≤0.1 cm, and ≤0.5 cm were evaluated. Associations with adverse histopathological features were examined using multivariable logistic regression. Overall survival (OS) and disease-free survival (DFS) were analyzed in 181 patients with available follow-up using Kaplan–Meier curves and Cox regression.

**Results:**

T-VPD ≤0.5 cm was independently associated with lymphovascular invasion, whereas T-VPD ≤0.05 cm correlated with poor differentiation and tumor deposits. In OS analysis, advanced age, nodal metastasis, lymphovascular invasion, and perineural invasion were adverse prognostic factors, while a strong peritumoral lymphocytic response was protective. Tumor deposits predicted worse DFS, and peritumoral lymphocytic response remained protective. T-VPD was not an independent predictor of survival.

**Conclusion:**

T-VPD reflects aggressive tumor behavior but lacks independent prognostic value. Combined with established adverse features, it may provide additional information for risk stratification in pT3 colon cancer.

## Introduction

Colorectal cancer (CRC) is among the most common malignancies worldwide and remains one of the leading causes of cancer-related mortality [1]. Despite advances in surgical techniques and adjuvant chemotherapy strategies, long-term survival rates in intermediate and advanced-stage disease remain suboptimal [2, 3]. In current clinical practice, prognosis is largely determined by the TNM staging system; however, because this system primarily reflects anatomical extent, it may not fully capture tumor biology in certain patient subgroups. T3 disease represents a typical example of this limitation: the depth of invasion beyond the muscularis propria shows considerable variability, leading to marked heterogeneity in clinical outcomes within the same T3 category [4].

For this reason, microscopic subclassification of T3 tumors has attracted increasing attention. Studies evaluating the relationship between the depth of extramural invasion and metastatic spread or survival suggest that microscopic distance may reflect aggressive tumor behavior. In this context, the tumor–visceral peritoneal distance may represent not only an anatomical measurement but also a parameter associated with histopathological features reflecting invasive tumor behavior.

Accordingly, this study aimed to investigate in pT3 colon adenocarcinoma:

1. The association of T-VPD with histopathological markers of aggressiveness (lymphovascular invasion, perineural invasion, tumor differentiation, and tumor deposits);
2. Its potential impact on overall and disease-free survival;
3. Clinically meaningful threshold values; and
4. Its applicability as a parameter contributing to risk stratification in pathological reporting.

This study was designed to further characterize the heterogeneity of pT3 colon adenocarcinoma and to evaluate the potential role of T-VPD as a complementary histopathological parameter associated with aggressive tumor features.

## Materials and methods

### Ethics statement

This study was approved by the Ethics Committee of Kafkas University Faculty of Medicine (decision no. KAÜ-TFEK 2025/07-17, session dated 24 September 2025). The study was conducted in a retrospective design using archived pathological materials and patient records. All patient data were anonymized, and no personal identifiers were included. The requirement for informed consent was waived by the ethics committee due to the retrospective nature of the study and the use of anonymized data. The study was conducted in accordance with the principles of the Declaration of Helsinki.

### Study design and cases

In this retrospective cross-sectional study, patients diagnosed with and surgically resected for pT3 colon adenocarcinoma between 2010 and 2022 were evaluated. A total of 391 cases were initially identified.

Cases in which the visceral peritoneum could not be reliably evaluated were excluded prior to analysis (n = 62). The exclusion criteria were as follows:

1. Inadequate tissue sampling with absent or fragmented serosal surface (n = 8);
2. Technical artifacts preventing assessment of the mesothelial lining (e.g., cautery distortion or tissue folding) (n = 2);
3. Tumors in which the deepest invasive focus was directed toward a non-peritonealized (retroperitoneal) surface, precluding reliable measurement of the tumor–visceral peritoneal distance (n = 52). In these cases, measurement relative to an alternative peritonealized surface was intentionally avoided to prevent systematic measurement bias.

Therefore, only tumors in which the deepest point of invasion was directed toward a peritonealized surface were included in the T-VPD analysis. All histopathological slides and pathology reports of the remaining 329 cases were re-evaluated. Clinicopathological data, including age, sex, tumor location and size, stage, recurrence status, and survival information, were recorded.

Analyses were conducted using two separate datasets:

1. The entire pT3 cohort for evaluation of histopathological associations (n = 329), and
2. The follow-up subcohort for survival analyses (n = 181).

The lack of follow-up data for the entire cohort resulted in survival analyses being restricted to a subset of patients (n = 181).

Importantly, only tumors in which the deepest invasive front was directed toward a peritonealized surface were eligible for T-VPD measurement. Consequently, the findings of the present study are applicable only to pT3 colon adenocarcinomas involving peritonealized surfaces and should not be extrapolated to tumors invading non-peritonealized (retroperitoneal) surfaces.

### Histopathological evaluation and measurement protocol

The tumor–visceral peritoneal distance (T-VPD) was defined as the shortest perpendicular distance from the deepest invasive tumor front to the true serosal surface and was measured in micrometers. Measurements were performed at ×10–×20 magnification after digital scale calibration of all slide images.

T-VPD measurements were independently performed by two pathologists. Interobserver agreement was assessed using a two-way random-effects intraclass correlation coefficient (ICC) based on absolute agreement.

Throughout the manuscript, the term “T-VPD” refers to the shortest distance between the invasive tumor front and the true serosal surface. For simplicity, the term “visceral peritoneum” is used throughout the text to indicate this anatomical serosal boundary, although the measurements were performed using the true serosal surface as the reference landmark. In cases showing reactive serositis or fibroinflammatory changes, displaced mesothelium was not accepted as the reference landmark; instead, the outer anatomical serosal collagenous boundary was used for measurement. Cases with a T-VPD of 0 µm were excluded from all T-VPD-based analyses because no measurable distance remained between the invasive tumor front and the serosal surface. This exclusion was applied *a priori* to ensure consistent quantitative assessment of T-VPD. In cases with multiple tumor-containing slides, the slide demonstrating the deepest invasive tumor front closest to the serosal surface was selected for measurement. When more than one slide was considered suitable, T-VPD was measured on the section showing the shortest distance between the invasive front and the true serosal surface.

For analytical purposes, tumor differentiation was dichotomized as well versus poor differentiation; moderately differentiated tumors were grouped with well-differentiated cases. Mucinous adenocarcinomas were excluded from differentiation analyses due to the limited prognostic relevance of conventional grading in this subtype.

### Distance classification approach

T-VPD was categorized into binary, three-level, and five-level groups to explore its association with tumor biology. This multilevel classification strategy was designed as an exploratory analysis based on the hypothesis that invasive histopathological patterns may vary according to threshold-based proximity to the visceral peritoneum. No correction for multiple testing was applied, and findings should be interpreted as hypothesis-generating rather than predefined clinical cut-off values. The identified threshold values require validation in independent cohorts before being considered clinically applicable.

### Survival data

Long-term follow-up data were available for 181 pT3 cases, and survival analyses were conducted within this subgroup. Accordingly, survival results represent only patients with available follow-up data rather than the entire cohort.

Within this subgroup, AJCC clinical stage distribution at diagnosis was as follows: 86 patients (47.5%) stage II, 74 patients (40.9%) stage III, and 21 patients (11.6%) stage IV.

During follow-up, 26 patients (14.4%) developed recurrence. At the end of the study period, 74 patients (40.9%) had died and 107 (59.1%) were alive. No imputation methods were applied for missing follow-up data. Patients presenting with distant metastasis at diagnosis were included in the overall cohort to reflect biological heterogeneity; however, they were excluded from disease-free survival (DFS) analyses. This represents a limitation in the interpretation of survival outcomes.

To assess potential selection bias, clinicopathological characteristics were compared between patients with available follow-up data and those without follow-up data. Among the 181 patients included in survival analyses, 140 (77.3%) received adjuvant chemotherapy and 32 (17.7%) demonstrated dMMR/MSI status.

### Statistical analysis

Statistical analyses were performed using SPSS version 26. Categorical variables were compared using the chi-square test or Fisher’s exact test, as appropriate.

Univariable Cox regression analyses were initially performed, followed by multivariable models. Variables included in multivariable analyses were selected based on established clinicopathological relevance, statistical significance in univariable analyses, and the need to maintain an events-per-variable ratio greater than 10 in order to reduce the risk of overfitting. Accordingly, only a limited number of biologically and clinically meaningful variables were retained in the final multivariable models. This approach was adopted to minimize model overfitting and preserve statistical stability. Independent associations between T-VPD and lymphovascular invasion (LVI), perineural invasion (PNI), tumor deposits, and differentiation were evaluated using multivariable logistic regression analysis.

Kaplan–Meier curves were generated for overall survival (OS) and disease-free survival (DFS), and groups were compared using the log-rank test. Cox proportional hazards regression was used to identify independent prognostic factors. The proportional hazards assumption was assessed using log-minus-log survival plots and no violations were observed. Due to the limited number of events in DFS analyses, multivariable Cox regression modeling was not performed for DFS.

Statistical significance was defined as p < 0.05. Results with p ≥ 0.05 were considered statistically non-significant. Exact p values are presented throughout the manuscript.

## Results

A total of 329 cases of pT3 colon adenocarcinoma were included in the study. Mean distance to the visceral peritoneum was 0.30 cm (3,000 μm; range: 50–16,000 µm). All measurements were performed in micrometers and converted to centimeters for statistical analyses. The mean age was 63 years, the mean tumor size was 4.6 cm, and the mean follow-up duration was 60.8 months. The clinical and macroscopic characteristics of the cases are presented in Table 1, and the histopathological characteristics are presented in Table 2.

**TABLE 1:** Demographic and tumor characteristics of the entire pT3 cohort (n = 329).

Tumor location	Right colon n (%)	Left colon n (%)
​	87 (26.4)	242 (73.6)
Age	≤50 years n (%)	>50 years n (%)
​	49 (14.9)	280 (85.1)
Sex	Female n (%)	Male n (%)
​	136 (41.3)	193 (58.7)
Tumor size	≤5 cm n (%)	>5 cm n (%)
​	217 (66.0)	112 (34.0)

All cases were histopathologically classified as pT3 colon adenocarcinoma. Percentages were calculated based on the total cohort (n = 329).

**TABLE 2:** Histopathological Characteristics of the entire pT3 cohort (n = 329).

Histopathological features	High n (%)	Intermediate n (%)	Low n (%)
PDC (poorly differentiated clusters)	129 (39.2)	107 (35.2)	93 (28.3)
Tumor budding (TB)	150 (45.6)	97 (29.5)	82 (24.9)
Histopathological features	High n (%)	Low n (%)	Absent n (%)
Intratumoral lymphocytic response	46 (14.0)	222 (67.5)	61 (18.5)
Peritumoral lymphocytic response	188 (57.1)	141 (42.6)	​

Histopathological Features	Well differentiated n (%)	Poorly differentiated n (%)
Differentiation	278 (84.5)	32 (9.7)
Histopathological features	Present n (%)	Absent n (%)
Lymphovascular invasion (LVI)	225 (68.4)	104 (31.6)
Perineural invasion (PNI)	108 (32.8)	221 (67.2)
Tumor deposits (TD)	36 (10.9)	293 (89.1)
Lymph node metastasis	170 (51.7)	159 (48.3)
Crohn-like lymphocytic reaction	210 (63.8)	119 (36.2)

All cases were histopathologically classified as pT3 colon adenocarcinoma. Percentages were calculated based on the total cohort (n = 329). Differentiation was assessed only in non-mucinous adenocarcinomas. Mucinous adenocarcinomas (n = 19) were excluded from differentiation-based analyses.

### Distance to the visceral peritoneum and histopathological aggressiveness

Tumors were classified according to various threshold values based on the assumption that T-VPD might be associated with tumor biological behavior at different distance levels, and their relationships with histopathological variables were analyzed. Interobserver agreement for T-VPD measurements was found to be excellent (ICC = 0.968; 95% CI: 0.960–0.974; p < 0.001). Classifications of the five separate groups are shown in Table 3.

**TABLE 3:** Stratification of tumor-visseral peritoneal distance by cut-off groups.

Group A	Category	Distance (cm)	Number of cases (n)	Percentage (%)
​	≤0.01 cm	0–0.01	6	1.8
	>0.01, ≤0.1	0.01–0.1	42	12.8
	>0.1, ≤0.5	0.1–0.5	247	75.1
	>0.5, ≤1	0.5–1	32	9.7
	>1 cm	>1	2	0.6

Group B	Category	Distance (cm)	Number of cases (n)	Percentage (%)
	≤0.5 cm	0–0.5	295	89.7
	>0.5 cm	>0.5	34	10.3

Group C	Category	Distance (cm)	Number of cases (n)	Percentage (%)
	≤0.05 cm	0–0.05	16	4.9
	>0.05 cm	>0.05	313	95.1

Group D	Category	Distance (cm)	Number of cases (n)	Percentage (%)
	≤0.05 cm	0–0.05	16	4.8
	>0.05, ≤0.1 cm	>0.05–0.1	32	9.8
	>0.1 cm	>0.1	281	85.4

Group E	Category	Distance (cm)	Number of cases (n)	Percentage (%)
	≤0.1 cm	0–0.1	48	14.6
	>0.1 cm	>0.1	281	85.4

Group F	Category	Distance (cm)	Number of cases (n)	Percentage (%)
	≤0.01 cm	0–0.01	6	1.8
	>0.01 cm	>0.01	323	98.2

In exploratory analyses, significant associations were concentrated around the 0.05 cm and 0.5 cm thresholds. In contrast, classifications based on the 0.01 cm and 0.1 cm cutoffs did not demonstrate consistent associations with histopathological parameters. Therefore, subsequent analyses focused on the ≤0.5 cm and ≤0.05 cm thresholds. These cutoffs should be interpreted as exploratory rather than predefined biological thresholds.

Tumors located closer to the visceral peritoneum were associated with more aggressive histopathological features. Specifically, when the tumor–visceral peritoneal distance was **≤** 0.5 cm, there was a significantly higher frequency of lymphovascular invasion (p = 0.002) and perineural invasion (p = 0.023). This association became more pronounced at shorter distances; when the distance was **≤** 0.05 cm, tumors showed a stronger association with poor differentiation (p = 0.009) and the presence of tumor deposits (p = 0.013). The histopathological findings associated with the distance thresholds are summarized in Table 4.

**TABLE 4:** Summary of distance thresholds and associated aggressive features.

Distance threshold	Associated aggressive features
≤ 0.5 cm	Increased frequency of lymphovascular invasion (LVI) and perineural invasion (PNI)
≤ 0.05 cm	Stronger association with poor differentiation and presence of tumor deposits (TD)
≤ 0.01 cm	Serosal inflammation and T4a-like behavior; due to limited case number, considered hypothesis-level only

#### Independent factors associated with microscopic distance to the visceral peritoneum

Variables considered clinically and biologically meaningful and found to be significantly associated with T-VPD in univariate analyses were included in the multivariable logistic regression analyses. Due to the low number of events and to preserve model stability, the number of variables included in the model was limited.

##### Factors associated with perineural invasion (PNI)

The associations between T-VPD and PNI were evaluated using binary T-VPD classification (Group B: ≤0.5 cm and >0.5 cm). In logistic regression analyses, the dependent variable was coded as absence of PNI; therefore, OR values <1 reflect factors associated with the presence of PNI.

In the multivariable logistic regression analysis demonstrating independent factors associated with PNI (Supplementary Table 1), lymph node positivity (N+) was found to be independently and statistically significantly associated with the presence of PNI (OR = 0.54; 95% CI: 0.33–0.89; p = 0.015). Similarly, the presence of lymphovascular invasion (LVI) was significantly associated with the presence of PNI (OR = 0.42; 95% CI: 0.22–0.78; p = 0.006).

A tumor–visceral peritoneal distance of ≤0.5 cm was associated with an increased likelihood of PNI; however, this association did not reach statistical significance (OR = 0.42; 95% CI: 0.15–1.16; p = 0.094).

When tumor budding was included in the model, it was independently associated with the presence of PNI (overall p = 0.047). However, in subgroup analyses, neither high-grade budding (OR = 0.59; 95% CI: 0.31–1.14; p = 0.117) nor moderate-grade budding (OR = 1.19; 95% CI: 0.57–2.48; p = 0.647) showed a statistically significant association with PNI compared with low-grade budding.

The presence of a peritumoral lymphocytic response was not independently associated with PNI (OR = 1.13; 95% CI: 0.68–1.87; p = 0.639).

##### Factors associated with lymphovascular invasion (LVI)

The associations between T-VPD and LVI were evaluated using binary T-VPD classification (Group B: ≤0.5 cm and >0.5 cm). A multivariable logistic regression analysis was performed to determine independent factors associated with lymphovascular invasion (LVI) (Supplementary Table 2). In the logistic regression analysis, the dependent variable was coded as absence of LVI; therefore, OR values <1 reflect factors associated with the presence of LVI.

As a result of the analysis, a tumor–visceral peritoneal distance of ≤0.5 cm was found to be independently and statistically significantly associated with the presence of LVI (OR = 0.33; 95% CI: 0.15–0.74; p = 0.007). Similarly, lymph node positivity (N+) was independently associated with the presence of LVI (OR = 0.54; 95% CI: 0.31–0.96; p = 0.035).

The presence of perineural invasion (PNI) showed a strong and statistically significant association with the presence of LVI (OR = 2.53; 95% CI: 1.34–4.78; p = 0.004). In contrast, the presence of a peritumoral lymphocytic response was not independently associated with LVI (OR = 1.44; 95% CI: 0.84–2.46; p = 0.182).

When poorly differentiated clusters (PDC) were included in the model as an overall variable, they demonstrated a statistically significant association with the presence of LVI (overall p = 0.001). In subgroup analyses, high-grade PDC was significantly associated with the presence of LVI compared with low-grade PDC (OR = 0.29; 95% CI: 0.15–0.57; p < 0.001). In contrast, intermediate-grade PDC was not significantly associated with LVI when compared with low-grade PDC (OR = 0.54; 95% CI: 0.29–1.02; p = 0.056).

Tumor differentiation (poor vs. well/moderate) and the presence of tumor deposits did not show an independent association with LVI (p = 0.201 and p = 0.665, respectively).

##### Factors associated with tumor deposits (TD)

The associations between T-VPD and tumor deposits (TD) were analyzed using binary T-VPD classification (Group C: ≤0.05 cm and >0.05 cm). A multivariable logistic regression analysis was performed to identify independent factors associated with tumor deposits (Supplementary Table 3).

As a result of the analysis, a tumor–visceral peritoneal distance of ≤0.05 cm demonstrated a strong and independent association with the presence of TD (OR = 6.20; 95% CI: 1.82–21.12; p = 0.004). This finding indicates an association between shorter T-VPD and the presence of tumor deposits.

The presence of a peritumoral lymphocytic response was identified as an independent protective factor against the development of TD (OR = 0.29; 95% CI: 0.13–0.62; p = 0.002).

The presence of lymphovascular invasion (LVI) showed a statistically significant and positive association with TD (OR = 2.51; 95% CI: 1.01–6.25; p = 0.047).

In contrast, no statistically significant association was identified between the presence of perineural invasion (PNI) and tumor deposits (OR = 1.44; 95% CI: 0.68–3.05; p = 0.335).

##### Factors associated with and degree of differentiation

A multivariable logistic regression analysis was performed to determine independent factors associated with poor differentiation (Supplementary Table 4). In this analysis, the dependent variable was defined as the presence of poor differentiation.

As a result of the analysis, a tumor–visceral peritoneal distance of ≤0.05 cm showed a statistically significant and independent association with the presence of poor differentiation (OR = 4.72; 95% CI: 1.29–17.30; p = 0.019).

High peritumoral lymphocytic infiltration (high vs. low) was identified as an independent protective factor against the development of poor differentiation (OR = 0.29; 95% CI: 0.11–0.76; p = 0.012).

When tumor budding was included in the model as an overall variable, it demonstrated a statistically significant association with poor differentiation (overall p = 0.001). In subgroup analyses, moderate-grade budding was significantly associated with poor differentiation (OR = 4.77; 95% CI: 1.32–17.18; p = 0.017), whereas the association between high-grade budding and poor differentiation was not statistically significant (OR = 0.41; 95% CI: 0.06–2.62; p = 0.346).

Lymph node positivity (N+) was not significantly associated with poor differentiation (OR = 1.86; 95% CI: 0.81–4.26; p = 0.142).

### Survival analyses

#### Endpoint definitions

Overall survival (OS) was defined as the time from the date of curative-intent surgical resection to death from any cause or the date of last follow-up. Patients who were alive at the last follow-up were censored on the date of their last clinical evaluation. Disease-free survival (DFS) was defined as the time from the date of surgical resection to the first documented recurrence (local or distant) or death (whichever occurred first); cases without recurrence were censored at the date of last follow-up. Patients with distant metastasis at the time of diagnosis were excluded from DFS analyses. Recurrence and survival status were verified through hospital electronic records and follow-up documentation.

A multivariable Cox regression model was constructed to include a limited number of clinically meaningful variables consistent with the number of events.

Hazard ratios (HR) and 95% confidence intervals (CI) were obtained from univariate Cox regression models. For variables containing more than two categories, HR values were calculated according to a predefined reference group.

Among the 181 patients with available follow-up data, 140 (77.3%) received adjuvant chemotherapy, while 32 (17.7%) demonstrated dMMR/MSI status.

#### Overall survival (OS)

##### Univariate cox regression analyses

In univariate Cox regression analysis, lymph node positivity (HR = 2.68; 95% CI: 1.64–4.38; p < 0.001), lymphovascular invasion (HR = 2.48; 95% CI: 1.42–4.31; p = 0.001), perineural invasion (HR = 2.23; 95% CI: 1.38–3.61; p = 0.001), the presence of tumor deposits (HR = 2.28; 95% CI: 1.16–4.49; p = 0.017), and low peritumoral lymphocytic infiltration (HR = 2.08; 95% CI: 1.29–3.37; p = 0.003) were identified as significant adverse prognostic factors for overall survival.

Age over 50 years was also associated with an increased risk of death (HR = 2.12; 95% CI: 1.00–4.47; p = 0.049). Left-sided colon location was associated with worse overall survival; however, this association did not reach statistical significance (HR = 1.80; 95% CI: 0.99–3.29; p = 0.055).

Sex, tumor size, histological differentiation, intratumoral lymphocytic infiltration, Crohn-like lymphocytic response, tumor budding, poorly differentiated clusters, adjuvant chemotherapy, MSI status and T-VPD groups defined by different cutoff values (≤0.5 cm and ≤0.05 cm) did not show a significant association with overall survival. Detailed univariate results are presented in Supplementary Table 5.

##### Multivariable cox regression analyses

To reduce the risk of overfitting, multivariable Cox regression analyses were restricted to a parsimonious model including a limited number of clinically meaningful variables, thereby ensuring that the events-per-variable ratio exceeded 10.

In the multivariable Cox regression analysis, lymphovascular invasion (LVI), perineural invasion (PNI), lymph node positivity, and low peritumoral lymphocytic response were identified as independent adverse prognostic factors for overall survival. The presence of LVI increased the risk of death by approximately 2.1-fold (HR = 2.15; 95% CI: 1.18–3.92; p = 0.013), PNI by 2.3-fold (HR = 2.33; 95% CI: 1.40–3.87; p = 0.001), and lymph node positivity by 2.3-fold (HR = 2.31; 95% CI: 1.40–3.81; p = 0.001). Similarly, low peritumoral lymphocytic response was associated with approximately 2.3-fold worse survival compared with cases showing high response (HR = 2.30; 95% CI: 1.41–3.75; p = 0.001).

Age ≥50 years was not significantly associated with overall survival (HR = 1.85; 95% CI: 0.89–3.84; p = 0.099).

T-VPD ≤0.5 cm was not independently associated with overall survival after adjustment for other clinicopathological variables (HR = 0.56; 95% CI: 0.28–1.11; p = 0.098).

The results of the multivariable Cox regression analysis related to overall survival are presented in Table 5. The overall significance test of the multivariable model was statistically significant (Omnibus χ^2^ = 47.91; df = 6; p < 0.001), indicating that the model demonstrated adequate overall fit.

**TABLE 5:** Multivariable Cox regression analysis for overall survival (OS) (Follow-up cohort, n = 181).

Variable	Compared group	Reference group	HR (Exp(B))	95% CI	p value
Age	≥50 years	<50 years	1.85	0.89–3.84	0.099
LVI	Present	Absent	2.15	1.18–3.92	0.013
PNI	Present	Absent	2.33	1.40–3.87	0.001
Nodal status	Positive	Negative	2.31	1.40–3.81	0.001
Peritumoral lymphocytic response	Low	High	2.30	1.41–3.75	0.001
T–VPD	≤0.5 cm	>0.5 cm	0.56	0.28–1.11	0.098

Hazard ratios (HRs) and 95% confidence intervals (CIs) were derived from multivariable Cox proportional hazards regression analysis. HR > 1 indicates increased risk of death.

##### Kaplan–Meier survival analysis

Kaplan–Meier survival analysis demonstrated that when threshold values of 0.5 cm (Group B) and 0.05 cm (Group C) were used for tumor–visceral peritoneal distance, there was no statistically significant difference in overall survival between patients located closer to or farther from the visceral peritoneum according to these cutoffs.

**FIGURE 1:**
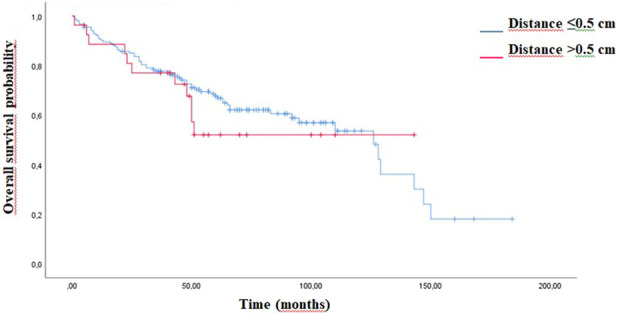
Kaplan-Meier overall survival according to tumor-visceral peritoneal distance (T-VPD). No statistically significant difference in overall survival was observed between tumors with T-VPD ≤0.5 cm and >0.5 cm (log-rank χ^2^ = 0.396, p = 0.529).

For the 0.5 cm threshold, log-rank χ^2^ = 0.396 (p = 0.529) (Figure 1), and for the 0.05 cm threshold, log-rank χ^2^ = 0.651 (p = 0.420). Censored cases were indicated with plus signs on the Kaplan–Meier curves.

##### Disease-free survival (DFS)

Disease-free survival (DFS) was evaluated only in patients without distant metastasis at diagnosis (M0). Patients with distant metastasis at diagnosis (M1) were excluded from DFS analyses, as they did not experience a disease-free interval by definition.

In the univariate Cox regression analysis for DFS, the presence of tumor deposits was found to be significantly associated with an increased risk of recurrence (HR = 4.1; 95% CI: 1.5–11.2; p = 0.005). Similarly, cases with low peritumoral lymphocytic infiltration had significantly worse disease-free survival compared with those showing high infiltration (HR = 2.9; 95% CI: 1.2–6.6; p = 0.015).

In contrast, tumor size, tumor budding, poorly differentiated clusters, intratumoral lymphocytic infiltration, lymph node status, lymphovascular invasion, perineural invasion, MSI status and adjuvant chemotherapy did not show a statistically significant association with DFS in univariate analyses. The univariate analysis results are presented in Supplementary Table 6.

Due to the limited number of events for DFS, multivariable Cox regression analysis was not performed in order to prevent model overfitting. Therefore, the DFS findings are exploratory in nature and should be interpreted as hypothesis-generating.

##### Kaplan–Meier survival analysis

Kaplan–Meier survival analysis showed that when 0.5 cm (Group B) and 0.05 cm (Group C) thresholds were used for tumor–visceral peritoneal distance, there was no statistically significant difference in disease-free survival between patients located closer to or farther from the visceral peritoneum according to these cutoffs.

**FIGURE 2:**
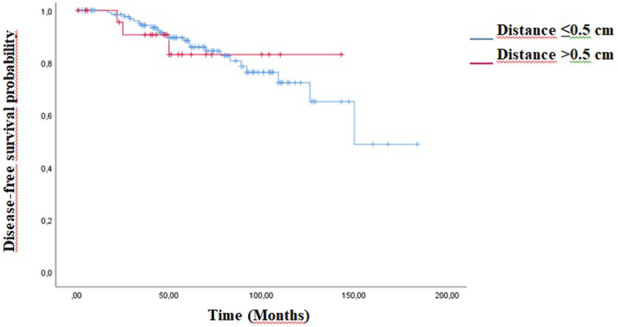
Kaplan-Meier analysis of disease-free survival according to tumor- visceral peritoneal distance (T-VPD). No statistically significant difference in disease-free survival was observed between tumors with T-VPD ≤0.5 cm and >0.5 cm (log-rank χ^2^ = 0.001, p = 0.975). Censored cases are indicated by plus signs.

For the 0.5 cm threshold, log-rank χ^2^ = 0.001 (p = 0.975) (Figure 2), and for the 0.05 cm threshold, log-rank χ^2^ = 0.062 (p = 0.803).

Comparison of patients with and without available follow-up data demonstrated generally similar clinicopathological characteristics. However, significant differences were observed in lymphovascular invasion, tumor budding grade, poorly differentiated clusters, and intratumoral lymphocytic infiltration, indicating that a degree of selection bias cannot be completely excluded (Supplementary Table 7).

## Discussion

Stage T3 colorectal cancers represent a tumor group exhibiting heterogeneous biological behavior due to the wide range of extension beyond the muscularis propria. Therefore, it has long been emphasized that a single morphological definition may not adequately reflect prognosis within the T3 category and that different histopathological and biological risk profiles exist within this group. In the literature, evaluation of T3 stage according to the depth of invasion beyond the muscularis propria has been shown to better predict the risk of lymph node metastasis and distant metastasis [5]. The study by Merkel et al., which proposed subdividing the pT3 stage according to different invasion depths, supports this approach [6]. Similarly, Arencibia-Pérez et al. reported that the depth of invasion into pericolic adipose tissue is an independent determinant of local and systemic recurrence as well as cancer-related mortality, and highlighted that a 5 mm threshold may be clinically critical [4]. Taken together, these studies indicate that although the T3 stage appears as a single anatomical category, it demonstrates a biologically heterogeneous spectrum of behavior.

The study by Pantaleon-Vasquez et al., reporting that tumors located ≤1 mm from the serosa but not macroscopically involving the serosal surface may exhibit histopathological and biological patterns similar to T4a cases, is one of the key findings emphasizing the biological importance of microscopic distance [7]. This study suggests that tumor proximity to the serosa may be associated with histopathological features reflecting invasive tumor behavior rather than representing a purely anatomical measurement.

In our study, analyses of both Group B (≤0.5 cm) and Group C (≤0.05 cm) demonstrated that different components of histopathological aggressiveness become prominent at different distance thresholds. At ≤0.5 cm, a marked increase in the frequency of lymphovascular invasion (LVI) and a trend toward increased perineural invasion (PNI) suggest that this distance range may be associated with invasion-related dissemination patterns. In contrast, the significant increase in poor differentiation and tumor deposits (TD) observed at ≤0.05 cm indicates that tumors located very close to the serosa may be accompanied by more aggressive histopathological features. However, given the exploratory nature of the threshold analyses, these observations should be interpreted cautiously and require validation in independent cohorts.

### Lymphovascular invasion (LVI) and perineural invasion (PNI)

In our study, multivariable analyses demonstrated that LVI was independently more frequent in tumors located ≤0.5 cm from the visceral peritoneum. PNI was not independently associated with T-VPD in multivariable analysis. These findings suggest that proximity to the visceral peritoneum may be more strongly associated with vascular invasion patterns.

Microscopic proximity of the tumor to the serosa may reflect a morphological context associated with more aggressive histopathological features and invasive growth patterns. In this framework, T-VPD appears to function primarily as a morphologic marker associated with tumor aggressiveness rather than as an independent biological determinant.

Both LVI and PNI are well-established adverse histopathological factors associated with aggressive tumor behavior, advanced stage, metastatic potential, and poorer survival outcomes in colorectal cancer [8–17].

In the literature, increasing proximity to the visceral peritoneum in colorectal cancers has been reported to be associated with higher frequencies of LVI and PNI [5, 11]. As stromal infiltration deepens, the likelihood of invasion into lymphatic and neural structures increases; LVI and PNI have been emphasized as correlating with invasion depth and as indicators of advanced stromal invasion [18, 19].

In our study, tumors located ≤0.5 cm from the visceral peritoneum demonstrated a significantly higher frequency of LVI. PNI accompanied this increase but did not retain independence in multivariable analyses, likely due to its strong association with other invasive parameters such as LVI and lymph node positivity. Furthermore, the independent associations of LVI with high PDC levels, lymph node positivity, and PNI indicate that these parameters frequently coexist in tumors with aggressive histopathological characteristics.

### Differentiation and tumor deposits (TD)

The degree of differentiation is an important component of T3 biology. Previous studies have reported that increased extramural spread is associated with poor differentiation, supporting the concept that more invasive tumors tend to exhibit more aggressive histopathological features [4, 6, 20].

In our study, both univariate and multivariable analyses revealed that tumors located ≤0.05 cm from the visceral peritoneum had a significantly higher rate of poor differentiation. This finding indicates that shorter T-VPD is associated with histopathological features of tumor aggressiveness, including poor differentiation. High peritumoral lymphocytic infiltration was identified as an independent protective factor against the development of poor differentiation. When tumor budding was included in the model as an overall variable, it demonstrated a significant association with poor differentiation, whereas lymph node positivity, although supportive of this relationship, did not reach statistical significance.

Tumor deposits (TD) are defined as discrete tumor foci within pericolic or perirectal adipose tissue lacking identifiable lymph node structure and are currently evaluated only under the N1c category in the TNM system [21]. However, accumulating evidence indicates that TD are prognostically important not only in node-negative cases but across all stages and may, in certain circumstances, represent a risk marker as strong as lymph node metastasis [21–23]. The presence of TD is associated with increased risk of liver, lung, and peritoneal metastases, as well as shorter overall survival (OS) and disease-free survival (DFS) [21, 24, 25].

Recent studies have shown that not only the presence but also the number of TD is directly associated with clinical outcomes. Lundström et al. reported that the number of TD has an independent impact on survival [26]. Moreover, the npN model, which evaluates TD similarly to metastatic lymph nodes, has been shown to improve prognostic accuracy compared with classical pN classification [27]. The “nN” approach (TD + number of positive lymph nodes) proposed by Zheng et al. has also been demonstrated to improve survival prediction [28]. These data clearly indicate that TD should be reconsidered within the TNM system.

Additionally, the frequent coexistence of TD with poor differentiation, LVI, and PNI supports its role as a marker of aggressive tumor behavior [24, 29, 30].

In conclusion, both univariate and multivariable analyses in our study demonstrated that tumors located ≤0.05 cm from the visceral peritoneum had a significantly higher frequency of TD, that TD frequency increased in the presence of LVI, and that a high peritumoral lymphocytic response was an independent protective factor against the development of TD. Taken together, these findings indicate that T-VPD alone is not a parameter that alters staging; however, when considered together with aggressive histopathological features such as poor differentiation, TD, LVI, and PNI, it may provide a microscopic risk profile within the T3 stage. This approach may constitute a complementary framework requiring future validation for more refined prognostic assessment and adjuvant treatment decisions within the T3 stage.

In addition, a high peritumoral lymphocytic response was independently associated with a lower frequency of both tumor deposits and poor differentiation in the present cohort.

### Survival analyses and biological model

The restriction of survival analyses to patients with available follow-up data (approximately 55% of the total cohort) represents an important limitation that may reduce the generalizability of survival outcomes and introduce potential survival bias. Therefore, survival findings should be interpreted independently from the histopathological analyses representing the entire cohort and with appropriate caution. In addition, although most clinicopathological characteristics were comparable between patients with and without available follow-up data, some differences were observed in lymphovascular invasion, tumor budding grade, poorly differentiated clusters, and intratumoral lymphocytic infiltration, suggesting the possibility of selection bias in survival analyses.

### Overall survival (OS)

Univariate Cox regression analyses identified advanced age, lymph node positivity, lymphovascular invasion (LVI), perineural invasion (PNI), tumor deposits, and low peritumoral lymphocytic infiltration as adverse prognostic factors for overall survival. Left-sided tumor location was associated with worse overall survival; however, this association did not reach statistical significance. In contrast, tumor size, histological differentiation, tumor budding, poorly differentiated clusters, and intratumoral lymphocytic infiltration were not significantly associated with overall survival.

In multivariable Cox regression analysis, lymph node positivity, LVI, PNI, and low peritumoral lymphocytic response were confirmed as independent adverse prognostic factors for overall survival. Notably, low peritumoral lymphocytic response was associated with approximately a 2.3-fold increased risk of death compared with cases showing high response, supporting an association between peritumoral lymphocytic response and survival outcomes.

T-VPD ≤0.5 cm was not significantly associated with overall survival (HR = 0.56; 95% CI: 0.28–1.11; p = 0.098). This finding suggests that the effect of T-VPD on survival should be interpreted not as a direct influence but rather in the context of its close association with aggressive invasive features such as LVI, PNI, and lymph node involvement. Indeed, shorter T-VPD was associated with several adverse histopathological features, particularly lymphovascular invasion, poor differentiation and tumor deposits. Taken together, these findings indicate that T-VPD may be more appropriately regarded as a complementary morphological marker associated with aggressive histopathological features rather than as an independent prognostic determinant.

### Disease-free survival (DFS)

Disease-free survival (DFS) analyses were restricted to patients without distant metastasis at diagnosis (M0). Since patients with metastatic disease (M1) do not experience a disease-free interval by definition, this approach is widely accepted in the literature and aims to ensure that DFS accurately reflects recurrence risk.

In univariate Cox regression analyses, the presence of tumor deposits (TD) was associated with a significant increase in recurrence risk. This finding is consistent with previous studies reporting that TD are associated with adverse oncological outcomes and increased recurrence risk [25]. The observed association between TD and DFS may reflect their relationship with aggressive tumor behavior.

Similarly, cases with low peritumoral lymphocytic infiltration had significantly worse disease-free survival compared with those showing high infiltration. This observation is consistent with the association of peritumoral lymphocytic infiltration with both overall survival and disease-free survival in the present cohort.

In contrast, tumor size, tumor budding, poorly differentiated clusters, intratumoral lymphocytic infiltration, lymph node status, LVI, and PNI did not show a statistically significant association with DFS in univariate analyses. This finding may reflect the limited number of DFS events and the restricted statistical power of the present cohort.

When evaluated using different threshold values (≤0.5 cm and ≤0.05 cm), T-VPD did not demonstrate a statistically significant association with DFS. This finding indicates that TD and peritumoral lymphocytic infiltration showed stronger associations with DFS than T-VPD in the present cohort.

Due to the limited number of events in DFS analyses, a multivariable Cox regression model could not be constructed. This limitation may have reduced statistical power for certain parameters. Therefore, larger, prospective, and adequately powered validation cohorts are required to more clearly determine the potential impact of T-VPD on DFS.

## Conclusion

This study demonstrates that in pT3 colon adenocarcinomas, tumor–visceral peritoneal distance (T-VPD) is not a prognostic marker on its own, but rather a complementary morphological parameter closely associated with histopathological features reflecting aggressive tumor biology. A decreasing T-VPD was associated with increased frequencies of lymphovascular invasion and perineural invasion at the ≤0.5 cm threshold, whereas independent associations were observed for lymphovascular invasion at this threshold and for poor differentiation and tumor deposits at the ≤0.05 cm threshold. These findings suggest that different T-VPD thresholds may be associated with different histopathological manifestations of tumor aggressiveness.

In survival analyses, T-VPD was not confirmed as an independent prognostic factor for either overall survival or disease-free survival. However, decreasing T-VPD showed significant associations with adverse histopathological parameters, particularly lymphovascular invasion, poor differentiation, and tumor deposits. These findings indicate that T-VPD is more closely associated with adverse histopathological features than with survival outcomes themselves. Furthermore, a high peritumoral lymphocytic response was associated with improved overall survival and disease-free survival in the present cohort.

In light of these findings, pT3 colon adenocarcinomas should not be regarded as a homogeneous clinical group; rather, they encompass distinct invasive and biological risk profiles at the microscopic level. Although T-VPD is not a criterion that independently alters the current TNM staging system, when evaluated together with histopathological parameters such as lymphovascular invasion, perineural invasion, tumor deposits, degree of differentiation, and immune response, it may contribute to a more refined risk stratification within the T3 stage.

In conclusion, there is currently insufficient evidence to support the routine use of T-VPD as a standard prognostic marker in pathological reporting. T-VPD may serve as a complementary component in future multiparametric prognostic models. Larger prospective studies and external validation cohorts are required to further clarify its clinical utility.

### Limitations

This study has several limitations. First, its retrospective single-center design and reliance on archival pathological material introduce the possibility of selection bias and may limit the generalizability of the findings.

Second, survival analyses were restricted to patients with available follow-up data (181 of 329 cases, approximately 55% of the total cohort). Comparison of patients with and without available follow-up data demonstrated differences in lymphovascular invasion, tumor budding grade, poorly differentiated clusters, and intratumoral lymphocytic infiltration. Therefore, the follow-up cohort may not fully represent the entire study population, and a degree of selection bias in survival analyses cannot be completely excluded.

Third, disease-free survival (DFS) analyses were limited to patients without distant metastasis at diagnosis (M0), as the concept of DFS does not apply to metastatic disease. In addition, the limited number of DFS events precluded multivariable Cox regression modeling, thereby reducing statistical power and restricting DFS analyses to the univariate level.

Fourth, the prognostic relevance of T-VPD was not validated in an independent cohort, and no internal validation procedures such as bootstrapping or cross-validation were performed. Consequently, the observed associations and threshold effects should be interpreted cautiously and require confirmation in external validation cohorts before clinical implementation.

Fifth, multiple T-VPD cutoff values were explored in an exploratory manner. No correction for multiple testing was applied; therefore, the identified thresholds (particularly 0.5 cm and 0.05 cm) should not be considered predefined clinical cutoff values but rather hypothesis-generating observations requiring further validation.

Sixth, although T-VPD measurements demonstrated excellent interobserver reproducibility (ICC >0.95), measurements were performed on retrospective tissue sections, and preanalytical variations related to tissue fixation, processing, and sectioning could not be fully controlled.

Finally, although adjuvant chemotherapy and MSI status were available and evaluated in survival analyses, other molecular alterations and cause-specific mortality data were not consistently available and therefore could not be incorporated into the analyses.

## Data Availability

The raw data supporting the conclusions of this article will be made available by the authors, without undue reservation.
