# Supplementary material for: Association between the histopathologic measurement of tumor–visceral peritoneal distance and prognosis in T3 colon adenocarcinoma
Source: Pathol Oncol Res. 2026 Jul 13;32:1612480. doi: 10.3389/pore.2026.1612480 (PMC13402222; doi:10.3389/pore.2026.1612480)
Supplement: Supplementary file 1 [file Table1.docx]

**Supplementary Table 1. Multivariable logistic regression analysis for factors associated with perineural invasion (PNI)**

| **Variable** | **Compared group** | **Reference group** | **OR (Exp(B))** | **95% CI** | **p value** |
| --- | --- | --- | --- | --- | --- |
| **Nodal stage** | **Positive** | **Negative** | **0.54** | **0.33–0.89** | **0.015** |
| **Lymphovascular invasion (LVI)** | **Present** | **Absent** | **0.42** | **0.22–0.78** | **0.006** |
| **Tumor–visceral peritoneal distance (T–VPD)** | **≤0.5 cm** | **>0.5 cm** | **0.42** | **0.15–1.16** | **0.094** |
| **Tumor budding (overall)** | **—** | **—** | **—** | **—** | **0.047** |
| **└ High grade** | **High** | **Low** | **0.59** | **0.31–1.14** | **0.117** |
| **└ Intermediate grade** | **Intermediate** | **Low** | **1.19** | **0.57–2.48** | **0.647** |
| **Peritumoral lymphocytic response** | **Present** | **Absent** | **1.13** | **0.68–1.87** | **0.639** |

**Multivariable logistic regression analysis was performed using the enter method. Variables were selected based on biological relevance and univariable analyses. Odds ratios (ORs) <1 reflect reference category coding. Statistical significance was defined as p < 0.05.**
