# Supplementary material for: Association between the histopathologic measurement of tumor–visceral peritoneal distance and prognosis in T3 colon adenocarcinoma
Source: Pathol Oncol Res. 2026 Jul 13;32:1612480. doi: 10.3389/pore.2026.1612480 (PMC13402222; doi:10.3389/pore.2026.1612480)
Supplement: Supplementary file 2 [file Table7.docx]

**Supplementary Table 7. Comparison of clinicopathological characteristics between patients with and without available follow-up data**

| **Variable** | **No follow-up (n=148)** | **Follow-up available (n=181)** | **p value** |
| --- | --- | --- | --- |
| **Sex** | | | 0.526 |
| Female | 64 (43.2%) | 72 (39.8%) |  |
| Male | 84 (56.8%) | 109 (60.2%) |  |
| **Age** | | | 0.525 |
| ≤50 years | 20 (13.5%) | 29 (16.0%) |  |
| >50 years | 128 (86.5%) | 152 (84.0%) |  |
| **Tumor location** | | | 0.472 |
| Right-sided | 42 (28.4%) | 45 (24.9%) |  |
| Left-sided | 106 (71.6%) | 136 (75.1%) |  |
| **Tumor size** | | | 0.398 |
| ≤5 cm | 94 (63.5%) | 123 (68.0%) |  |
| >5 cm | 54 (36.5%) | 58 (32.0%) |  |
| **Lymph node metastasis** | | | 0.735 |
| Negative | 70 (47.3%) | 89 (49.2%) |  |
| Positive | 78 (52.7%) | 92 (50.8%) |  |
| **Lymphovascular invasion** | | | **0.036** |
| Absent | 38 (25.7%) | 66 (36.5%) |  |
| Present | 110 (74.3%) | 115 (63.5%) |  |
| **Perineural invasion** | | | 0.420 |
| Absent | 96 (64.9%) | 125 (69.1%) |  |
| Present | 52 (35.1%) | 56 (30.9%) |  |
| **Tumor deposits** | | | 0.522 |
| Absent | 130 (87.8%) | 163 (90.1%) |  |
| Present | 18 (12.2%) | 18 (9.9%) |  |
| **Crohn-like lymphoid reaction** | | | 0.902 |
| Absent | 53 (35.8%) | 66 (36.5%) |  |
| Present | 95 (64.2%) | 115 (63.5%) |  |
| **Peritumoral lymphocytic response** | | | 0.150 |
| High | 91 (61.5%) | 97 (53.6%) |  |
| Low | 57 (38.5%) | 84 (46.4%) |  |
| **Differentiation** | | | 0.837 |
| Well/Moderate | 125 (89.3%) | 153 (90.0%) |  |
| Poor | 15 (10.7%) | 17 (10.0%) |  |
| **MSI status** | | | 0.076 |
| MSS/pMMR | 109 (73.6%) | 148 (81.8%) |  |
| MSI/dMMR | 39 (26.4%) | 33 (18.2%) |  |
| **Tumor budding** | | | **<0.001** |
| High | 76 (51.4%) | 74 (40.9%) |  |
| Intermediate | 58 (39.2%) | 39 (21.5%) |  |
| Low | 14 (9.5%) | 68 (37.6%) |  |
| **Poorly differentiated clusters (PDC)** | | | **<0.001** |
| High | 62 (41.9%) | 67 (37.0%) |  |
| Intermediate | 59 (39.9%) | 48 (26.5%) |  |
| Low | 27 (18.2%) | 66 (36.5%) |  |
| **Intratumoral lymphocytic infiltration** | | | **<0.001** |
| High | 31 (20.9%) | 15 (8.3%) |  |
| Low | 105 (70.9%) | 117 (64.6%) |  |
| Absent | 12 (8.1%) | 49 (27.1%) |  |

Differentiation was assessed only in non-mucinous adenocarcinomas. Mucinous adenocarcinomas (n = 19) were excluded from differentiation-based analyses.
