# Supplementary material for: Association between the histopathologic measurement of tumor–visceral peritoneal distance and prognosis in T3 colon adenocarcinoma
Source: Pathol Oncol Res. 2026 Jul 13;32:1612480. doi: 10.3389/pore.2026.1612480 (PMC13402222; doi:10.3389/pore.2026.1612480)
Supplement: Supplementary file 3 [file Table2.docx]

| **Supplementary Table 2. Multivariable logistic regression analysis for factors associated with lymphovascular invasion (LVI)** |
| --- |
| \| **Variable** \| **Compared group** \| **Reference group** \| **OR (Exp(B))** \| **95% CI** \| **p value** \| \| --- \| --- \| --- \| --- \| --- \| --- \| \| **Tumor–visceral peritoneal distance (T–VPD)** \| **≤0.5 cm** \| **>0.5 cm** \| **0.33** \| **0.15–0.74** \| **0.007** \| \| **Nodal stage** \| **Positive (N+)** \| **Negative** \| **0.54** \| **0.31–0.96** \| **0.035** \| \| **Perineural invasion (PNI)** \| **Present** \| **Absent** \| **2.53** \| **1.34–4.78** \| **0.004** \| \| **Peritumoral lymphocytic response** \| **Present** \| **Absent** \| **1.44** \| **0.84–2.46** \| **0.182** \| \| **Poorly differentiated clusters (PDC) – overall effect** \| **—** \| **—** \| **—** \| **—** \| **0.001** \| \| **└ High grade** \| **High** \| **Low** \| **0.29** \| **0.15–0.57** \| **<0.001** \| \| **└ Intermediate grade** \| **Intermediate** \| **Low** \| **0.54** \| **0.29–1.02** \| **0.056** \| \| **Tumor differentiation** \| **Poor** \| **Well/Moderate** \| **0.47** \| **0.14–1.50** \| **0.201** \| \| **Tumor deposits (TD)** \| **Present** \| **Absent** \| **0.79** \| **0.27–2.29** \| **0.665** \| |
| **Multivariable logistic regression analysis was performed using the enter method. Variables included in the model were selected based on biological relevance and univariable analyses. The dependent variable was coded as absence of lymphovascular invasion (reference category); therefore, OR values <1 indicate factors associated with the presence of lymphovascular invasion. Statistical significance was set at p < 0.05** |
