# Supplementary material for: Association between the histopathologic measurement of tumor–visceral peritoneal distance and prognosis in T3 colon adenocarcinoma
Source: Pathol Oncol Res. 2026 Jul 13;32:1612480. doi: 10.3389/pore.2026.1612480 (PMC13402222; doi:10.3389/pore.2026.1612480)
Supplement: Supplementary file 4 [file Table5.docx]

## Supplementary Table 5. Univariable Cox regression analysis for overall survival (OS)

*(Follow-up cohort, n = 181)*

| **Variable** | **Compared group** | **Reference group** | **HR (Exp(B))** | **95% CI** | **p value** |
| --- | --- | --- | --- | --- | --- |
| Sex | Male | Female | 0.97 | 0.61–1.55 | 0.899 |
| Age | >50 years | ≤50 years | 2.12 | 1.00–4.47 | **0.049** |
| Tumor location | Left colon | Right colon | 1.80 | 0.99–3.29 | 0.055 |
| Tumor size | >5 cm | ≤5 cm | 0.93 | 0.56–1.52 | 0.757 |
| Differentiation | Poor | Well/Moderate | 1.12 | 0.55–2.27 | 0.754 |
| Nodal status | Positive | Negative | 2.68 | 1.64–4.38 | **<0.001** |
| LVI | Present | Absent | 2.48 | 1.42–4.31 | **0.001** |
| PNI | Present | Absent | 2.23 | 1.38–3.61 | **0.001** |
| Tumor deposits (TD) | Present | Absent | 2.28 | 1.16–4.49 | **0.017** |
| Peritumoral lymphocytic infiltration | Low | High | 2.08 | 1.29–3.37 | **0.003** |
| Intratumoral lymphocytic infiltration | Moderate | High | 0.69 | 0.29–1.63 | 0.395 |
|  | Low | High | 1.21 | 0.50–2.92 | 0.680 |
| Crohn like lymphocytic response | Present | Absent | 1.01 | 0.63-1.62 | 0.96 |
| Tumor budding | Moderate | High | 1.42 | 0.85–2.38 | 0.178 |
|  | Low | High | 1.10 | 0.57–2.11 | 0.773 |
| PDC | Moderate | High | 1.16 | 0.69–1.98 | 0.575 |
|  | Low | High | 1.05 | 0.58–1.90 | 0.861 |
| Adjuvant chemotherapy | Present | Absent | 1.41 | 0.84–2.37 | 0.197 |
| MSI status | dMMR | MSS | 0.73 | 0.40–1.33 | 0.301 |
| T-VPD (Group B) | ≤0.5 cm | >0.5 cm | 0.81 | 0.43–1.55 | 0.531 |
| T-VPD (Group C) | ≤0.05 cm | >0.05 cm | 1.51 | 0.55-4.16 | 0.424 |

Hazard ratios (HRs) and 95% confidence intervals (CIs) were derived from Cox proportional hazards regression models. HR >1 indicates increased risk of death.
