# Supplementary material for: Association between the histopathologic measurement of tumor–visceral peritoneal distance and prognosis in T3 colon adenocarcinoma
Source: Pathol Oncol Res. 2026 Jul 13;32:1612480. doi: 10.3389/pore.2026.1612480 (PMC13402222; doi:10.3389/pore.2026.1612480)
Supplement: Supplementary file 5 [file Table3.docx]

**Supplementary Table 3. Multivariable Logistic Regression Analysis for Factors Associated with Tumor Deposits (TD)**

| **Variable** | **Compared group** | **Reference group** | **OR (Exp(B))** | **95% CI** | **p value** |
| --- | --- | --- | --- | --- | --- |
| **T–VPD (Group C)** | **≤0.05 cm** | **>0.05 cm** | **6.20** | **1.82–21.12** | **0.004** |
| **Peritumoral lymphocytic response** | **Present** | **Absent** | **0.29** | **0.13–0.62** | **0.002** |
| **Lymphovascular invasion (LVI)** | **Present** | **Absent** | **2.51** | **1.01–6.25** | **0.047** |
| **Perineural invasion (PNI)** | **Present** | **Absent** | **1.44** | **0.68–3.05** | **0.335** |

**Multivariable logistic regression analysis was performed using the enter method. Variables included in the model were selected based on biological relevance and univariable analyses. The dependent variable was coded as presence of tumor deposits (TD). Odds ratios (ORs) >1 indicate increased likelihood of tumor deposits. Statistical significance was defined as p < 0.05.**
