# Supplementary material for: Association between the histopathologic measurement of tumor–visceral peritoneal distance and prognosis in T3 colon adenocarcinoma
Source: Pathol Oncol Res. 2026 Jul 13;32:1612480. doi: 10.3389/pore.2026.1612480 (PMC13402222; doi:10.3389/pore.2026.1612480)
Supplement: Supplementary file 7 [file Table6.docx]

### Supplementary Table 6. Univariable Cox regression analysis of disease-free survival

| **Variable** | **Compared group** | **Reference group** | **HR (Exp(B))** | **95% CI** | **p value** |
| --- | --- | --- | --- | --- | --- |
| Tumor size | >5 cm | ≤5 cm | 1.7 | 0.77–3.6 | 0.200 |
| Tumor budding | Moderate | High | 0.54 | 0.22–1.33 | 0.181 |
|  | Low | High | 0.43 | 0.12–1.48 | 0.180 |
| PDC | Moderate | High | 0.58 | 0.23–1.44 | 0.241 |
|  | Low | High | 0.57 | 0.20–1.60 | 0.288 |
| Intratumoral lymphocytes | Moderate | High | 1.28 | 0.17–9.80 | 0.811 |
|  | Low | High | 2.26 | 0.30–17.76 | 0.436 |
| Peritumoral lymphocytes | Low | High | 2.9 | 1.2–6.6 | **0.015** |
| Tumor deposits | Present | Absent | 4.1 | 1.5–11.2 | **0.005** |
| N stage | Positive | Negative | 1.74 | 0.79–3.8 | 0.171 |
| LVI | Present | Absent | 1.6 | 0.7–3.7 | 0.260 |
| PNI | Present | Absent | 1.5 | 0.6–3.6 | 0.372 |
| Adjuvant chemotherapy | Present | Absent | 0.73 | 0.44–1.21 | 0.217 |
| MSI status | dMMR | MSS | 0.74 | 0.4–1.36 | 0.334 |
| T-DVP (Group B) | ≤0.5 cm | >0.5 cm | 0.98 | 0.3-3.3 | 0.975 |
| T-DVP (Group C) | ≤0.05 cm | >0.05 cm | 1.3 | 0.2-9.7 | 0.8 |

Hazard ratios (HRs) and 95% confidence intervals (CIs) were derived from univariable Cox proportional hazards regression models. HR >1 indicates increased risk of recurrence or death.
